# Daptomycin-Induced Pulmonary Toxicity: A Case Series

**DOI:** 10.7759/cureus.39613

**Published:** 2023-05-28

**Authors:** Yamini I Patel, Sarasija Natarajan, Srinivasarao Ramakrishna, Pius Ochieng

**Affiliations:** 1 Internal Medicine, The Wright Center for Graduate Medical Education, Scranton, USA; 2 Pulmonary and Critical Care Medicine, Geisinger Commonwealth School of Medicine, Scranton, USA

**Keywords:** antibiotic, bronchoscopy, eosinophilia, hypoxia, fever, pulmonary toxicity, adverse effects of daptomycin, drug induced eosinophilic pneumonia, eosinphilic pneumonia, daptomycin

## Abstract

Daptomycin is a bactericidal antibiotic used to treat methicillin-resistant Staphylococcus aureus (MRSA) and vancomycin-resistant enterococcus (VRE). Eosinophilic pneumonia is an uncommon but significant adverse effect of daptomycin. We present two patients treated with daptomycin who subsequently developed eosinophilic pneumonia (EP).

## Introduction

Daptomycin is a cyclic lipopeptide antibiotic that is bactericidal [1]. It was first approved by the United States Food and Drug Administration (FDA) in 2003 and only a limited number of pulmonary daptomycin toxicity cases resulting in acute eosinophilic pneumonia (AEP) have been reported since [2]. Diagnostic criteria for eosinophilic pneumonia (EP) secondary to daptomycin include exposure to daptomycin, fever, dyspnea, hypoxia, infiltrates on chest imaging, pulmonary eosinophilia (bronchoalveolar lavage (BAL) eosinophil count 25%), and clinical improvement after discontinuation of daptomycin [3].

## Case presentation

Case 1

A 74-year-old non-smoker female with a history of peripheral vascular disease, systemic hypertension, prior pulmonary embolism, dyslipidemia, and psoriatic arthritis presented with progressive shortness of breath, lethargy, and weakness for two days. She had been discharged from the hospital eight days prior, at which time she had been treated for osteomyelitis of the left femur and methicillin-resistant Staphylococcus aureus (MRSA) bacteremia with daptomycin at a dose of 10 mg/kg/day for six weeks, having had vancomycin stopped secondary to acute kidney injury. She had received 13 days of daptomycin at the time of presentation. She had no other new medication or supplement. In the emergency department (ED), the patient was hypoxemic with oxygen saturation of 90% on 3 liters per minute (lpm) of supplemental oxygen via nasal cannula. She was afebrile and hemodynamically stable but ill-appearing with moderate respiratory distress. She had decreased air entry bilaterally and absent wheeze with a normal heart exam and no cardiac murmur. There was left lower extremity with mild edema, clean surgical incision on the left thigh at the site of the abscess and the rest of the examination was unremarkable.

Investigations

**Figure 1:**
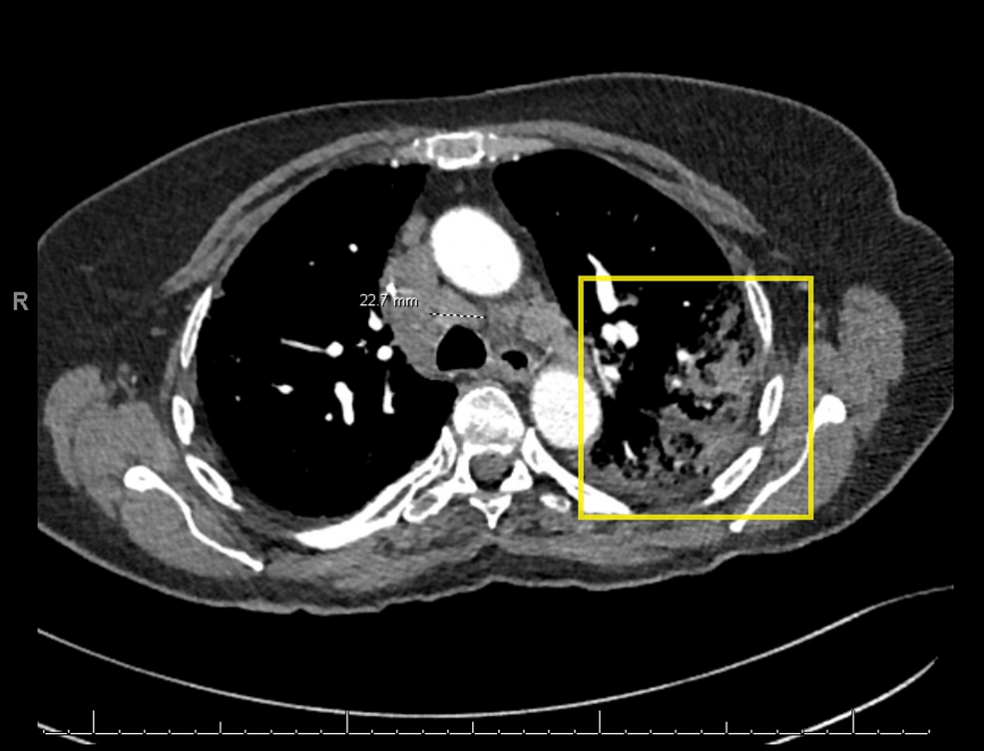
Computed tomography of the chest with contrast showing left lung infiltrates (yellow box) and mediastinal adenopathy (dotted black and white line)

Laboratory testing including a complete blood count (hemoglobin: 9.5 g/dL; white cells: 9.32 K/uL with absolute eosinophils 0.35 K/uL (3.8%); platelets: 233 K/uL; serum biochemistries (sodium: 136 mmol/L; potassium: 3.2 mmol/L; chloride: 100 mmol/L; bicarbonate: 23 mmol/L; blood urea nitrogen (BUN): 21 mg/dL; creatinine: 1.5 mg/dL, which was elevated from a baseline of 0.8 mg/dL but reduced compared to discharge creatinine 1.8 mg/dL; glucose: 110 mg/dL), troponin (high sensitivity troponin T: 22 ng/L), creatine kinase level 25 U/L, procalcitonin 0.52 ng/mL, and brain natriuretic peptide (BNP) 286 pg/mL. Respiratory virus polymerase chain reaction (PCR), including testing for COVID-19, was negative. Streptococcus pneumoniae and Legionella antigen were negative. Blood cultures showed no growth. Chest radiography (CXR) demonstrated retrocardiac opacity and faint airspace opacity in the left upper lung. Computed Tomography (CT) angiogram of the chest ruled out pulmonary embolism but was notable for small consolidations of the right lower lobe, and large posterior consolidations in the left lung reported as likely pneumonic infiltrates (Figure 1). A transthoracic echocardiogram (TTE) revealed no structural or valvular abnormalities and a left ventricular ejection fraction (LVEF) of 55%.

Treatment

The patient received a single dose of 40 mg oral prednisone in the ED, which was discontinued due to low suspicion of airway reactive disease. Antibiotic therapy with intravenous (IV) piperacillin-tazobactam was initiated in the ED given the possibility of pneumonia. Linezolid was instituted in place of daptomycin to continue treatment for the recent MRSA bacteremia. The piperacillin-tazobactam was then discontinued the next day with a decision to monitor clinically since there was a very low suspicion for pneumonia based on lack of fever, productive cough, and normal white blood cell count.

Outcome and Follow-Up

The patient was transitioned from 3 to 2 lpm on a nasal cannula. She was discharged to a skilled nursing facility on oral linezolid and oxygen supplementation. The pulmonary infiltrates resolved once daptomycin was stopped and eosinophils decreased from 0.35 K/uL on admission to 0.24 K/uL on discharge.

Case 2

A 77-year-old male with a medical history of coronary artery disease, systolic heart failure, hypertension, diabetes mellitus, chronic kidney disease stage 3a, and stroke, presented with progressive dyspnea for three days. He had been discharged from the hospital 13 days prior following treatment of osteomyelitis of the toe with amputation and daptomycin at a dose of 6 mg/kg/day for six weeks, of which he had received 17 days at the time of presentation. On presentation, he was hypoxemic requiring supplemental oxygen via nasal cannula at 6 lpm, tachycardic with 109 beats per minute, respiratory rate 18 breaths per minute, otherwise afebrile and hemodynamically stable. He had scattered bilateral rhonchi, a normal heart exam, bilateral lower extremity edema with decreased pulses, and a left foot wound deep to the tendon with surrounding erythema.

Investigations

**Figure 2:**
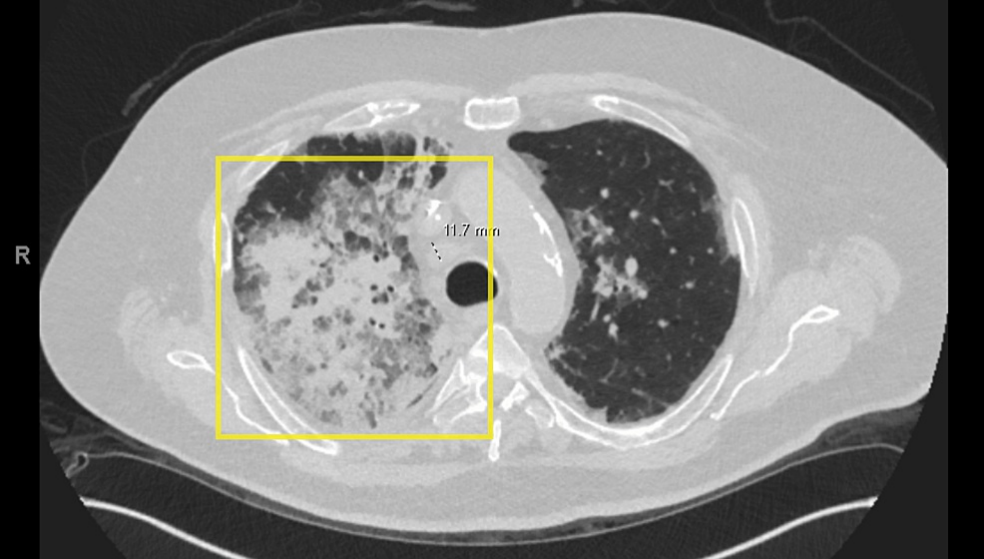
Computed tomography of the chest without contrast showing extensive right-sided infiltrates (yellow box) and hilar adenopathy (dotted black and white line) with smaller left-sided infiltrates

Laboratory testing including a complete blood count (hemoglobin: 9.9 g/dL; white cells: 14.67 K/uL with absolute eosinophils 0.6 K/uL (4.1%) that increased to 1.24 K/uL (8.4%) over the next 48 hours (normal: less than 0.5 K/uL or 1% to 6%); platelets: 652 K/uL; serum chemistries (sodium: 136 mmol/L; potassium: 4.1 mmol/L; chloride: 98 mmol/L; bicarbonate: 26 mmol/L; BUN: 49 mg/dL; creatinine: 1.6 mg/dL; glucose: 145 mg/dL), creatine kinase level 197 U/L, BNP was elevated to 8217 pg/mL which was noted to be a chronic elevation. C-reactive protein 460mg/L and erythrocyte sedimentation rate 95 mm/hour. Respiratory viral PCR including COVID-19, Streptococcus pneumoniae and Legionella urine antigen were negative. Blood cultures yielded no growth. Computed tomography of the chest showed extensive consolidating bilateral pulmonary opacities (Figure 2). TTE was notable for LVEF of 35% and thinning of the left ventricular wall which was unchanged from prior TTE. Bronchoscopy was done on hospital day six after receiving 80 mg methylprednisolone every six hours for two days, and BAL of the right middle lobe was notable for reactive pneumocytes and pulmonary macrophages in a background of inflammation with increased eosinophils 16%, negative for pneumocystis. Cultures of BAL yielded no pathogen/bacteria.

Treatment

The patient was started on IV piperacillin-tazobactam, vancomycin, and azithromycin; daptomycin was discontinued on admission. Azithromycin was discontinued after urine Legionella antigen was negative. IV methylprednisolone 80 mg was administered every six hours starting on the fourth day of admission, decreased to IV methylprednisolone 60 mg daily on day nine of hospitalization due to uncontrolled hyperglycemia despite insulin drip and further decreased to IV methylprednisolone 40 mg daily on day 13. The total duration of steroid treatment was 16 days.

Outcome and Follow-Up

The patient continued to deteriorate clinically requiring increased oxygen supplementation and was intubated on day seven of hospitalization. The hospital course was complicated by Clostridium difficile colitis treated with oral vancomycin for 10 days. CXR initially demonstrated improving infiltrate with steroids however repeat CXRs showed worsening bilateral infiltrates. Peripheral eosinophilia of 1.24 K/uL (8.4%) resolved with the administration of steroids. The patient was extubated to a high-flow nasal cannula after eight days of mechanical ventilation and supportive care but required re-intubation. He subsequently had a cardiac arrest and died on day 19 of hospitalization.

## Discussion

Daptomycin is an antimicrobial with activity against both aerobic and anaerobic gram-positive bacteria. It acts by calcium-dependent depolarization of bacterial cell membrane causing a release of intracellular potassium and cell death. It is used to treat MRSA and vancomycin-resistant enterococcus (VRE) skin and soft tissue infections, infective endocarditis, osteoarticular infections, and bacteremia [4]. It is avoided in the treatment of bronchoalveolar pneumonia due to its inactivation by alveolar surfactant [5]. Adverse effects of daptomycin include but are not limited to myopathy, drug reaction with eosinophilia and systemic symptoms (DRESS) syndrome, nausea/vomiting, neuropathy, neutropenia, daptomycin eosinophilic pneumonia (DEP), and tubulointerstitial nephritis [6]. EP secondary to daptomycin is uncommon. A review of case reports in 2017 found 32 reported cases between 1990 and 2017. Nonsteroidal anti-inflammatory drugs, ampicillin, nitrofurantoin, sulfonamides, minocycline, angiotensin-converting enzyme inhibitors, beta-blockers, hydrochlorothiazide, amiodarone, methotrexate, and many other medications have been shown to cause EP [7]. The mechanism and pathophysiology of DEP remain unknown. Proposed mechanisms include T-cell lymphocyte activation leading to the release of interleukin-5 and eotaxin causing eosinophil production and migration to the lungs, and inflammation and cell damage from the binding of daptomycin to pulmonary surfactant [8]. 

Three types of daptomycin eosinophilic syndromes have been described by West et al.: peripheral eosinophilia, primary DEP about four weeks into therapy, and DEP related to re-exposure. Elevated peripheral eosinophilia ≥5% was a risk factor for both primary and re-exposure DEP in a retrospective analysis of 330 patients who received daptomycin [9]. The percentage of patients with peripheral eosinophilia was found to be significantly higher in patients with DEP as were the leukocyte counts and C-reactive protein values [10]. A comprehensive systematic review by Uppal et al. estimated that the onset of fever and dyspnea occurred approximately 2.8 weeks following the initiation of daptomycin therapy at a dose of 4-10 mg/kg/day [11]. Among 40 cases of DEP described by Hirai et al., 73% received daptomycin dosage >6 mg/kg/day for a median of 14.8 days with the remaining having received <6 mg/day for a median of 23 days [12]. Soldevila-Boixader et al. analyzed the risk factors for DEP among patients with osteoarticular infection and proposed them to be age ≥70 years, therapy >14 days, and total cumulative dose of daptomycin ≥10 g. The total cumulative dose of daptomycin (TCDD) which is a product of the dose and length of therapy is considered a better estimate of the risk for DEP. TCDD >10 g is attained within two weeks of treatment with high-dose daptomycin [10]. Area under the concentration time curve (AUC) >939 mg.h/L is a similar measure used by Pham et al. as a measure of the risk for DEP. They recommend targeting a therapeutic AUC range of 666 to 939 mg.h/L to minimize the risk of adverse effects [3]. Our patients had TCDD of 9.7 gm and 12.7 gm respectively.

Another interesting cohort is the patients on hemodialysis (HD). Patients on HD who received >9 mg/kg of daptomycin were significantly associated with DEP [13]. In patients on HD, mortality with vancomycin treatment was significantly lower compared to patients on daptomycin despite similar clinical and microbiological effectiveness. Administering a loading dose of daptomycin was proposed to balance the disparity in mortality [14]. The diagnostic criteria for DEP, as proposed by the FDA, include fever, dyspnea with increasing oxygen requirements, new infiltrates on CXR or CT scan, concurrent exposure to daptomycin, >25% eosinophils on BAL, and clinical improvement with the withdrawal of daptomycin. The onset of fever, dyspnea, and hypoxia is usually between two to four weeks of exposure to daptomycin but can manifest sub-acutely as well [15]. Chest radiographs typically demonstrate pulmonary infiltrates, as seen in both of our patients. Other findings include diffuse bilateral ground-glass opacities, pleural effusions, and subpleural reticulonodular infiltrates. The Swordsman and Swartz criteria include additional details of eosinophilia on lung biopsy, exclusion of other causes of eosinophilia and recurrence of EP with re-exposure which despite its diagnostic utility could be potentially harmful. A review of the diagnostic criteria proposes a cut-off of 25% eosinophilia in BAL to be restrictive and invasive, in place of which a substitution with peripheral eosinophilia was suggested. A comparison of lung biopsy and BAL revealed eosinophilia in cases where BAL eosinophils were <25% implying that 25% might be too harsh a limit [3]. Kim et al. stratified 77 cases of DEP reported on the Adverse Event Reporting System database into definite, probable, possible, and unlikely. Definite was described as meeting all six FDA criteria, probable as BAL eosinophilia not exceeding 25% or the presence of only peripheral eosinophilia in the absence of fever, possible as an absence of fever, dyspnea, and eosinophilia, and unlikely as cases that did not meet any of the other criteria. All these categories were found to have patients with high pathological, biological, and temporal plausibility of DEP indicating that the criteria are not exclusive [16]. The cases we present had a relatively rapid onset of symptoms and met five of six of the FDA criteria for DEP. Our second case did have peripheral eosinophilia, however, did not meet BAL criteria of >25% eosinophils. We postulate that the lower yield of 16% eosinophils from the BAL is due to the high dose of steroids that the patient received. Despite neither of our cases meeting all six FDA criteria, the likelihood of DEP remains high. It is therefore reasonable to circumvent the use of invasive testing with BAL when a patient meets criteria for possible, probable and define DEP. 

The mainstay of treatment is the discontinuation of daptomycin and supportive care. Symptom improvement is commonly seen within 24 hours to one week of daptomycin discontinuation. The role of steroids remains controversial despite their ability to cause eosinophilic apoptosis [11]. Improvement of clinical symptoms solely with the discontinuation of daptomycin, without steroids, has been noted in a few patients [6]. Our first case received only one dose of steroids but showed clinical improvement with the discontinuation of daptomycin. However, there are also several reports illustrating the cases of patients in whom the use of steroids proved beneficial. Some regimens of steroids outlined include intravenous methylprednisolone 60-125 mg every six hours followed by tapering of oral prednisone 40-60 mg daily over two to six weeks has been used in some patients. A shorter course of two to four weeks of corticosteroid treatment has been shown to result in similar clinical and radiological outcomes [17]. Some cases of chronic steroid dependence have also been described in literature [7]. Our second case was managed with steroids and prompt discontinuation of daptomycin but continued to worsen requiring mechanical ventilation and succumbing to death indicating that the risk versus benefit of steroids is likely influenced by pre-existing patient factors and comorbidities. The role of steroids in the treatment of DEP needs to be explored further to enable the outlining of definitive management guidelines. 

## Conclusions

Timely recognition of daptomycin toxicity is necessary in a patient with clinical symptoms and imaging findings of eosinophilic pneumonia in the setting of daptomycin use. Discontinuation of daptomycin and supportive care remains the definitive management strategy pending further exploration of the role of glucocorticoids.
